# Oral Glucose Load and Human Cutaneous Microcirculation: An Insight into Flowmotion Assessed by Wavelet Transform

**DOI:** 10.3390/biology10100953

**Published:** 2021-09-23

**Authors:** Henrique Silva, Jernej Šorli, Helena Lenasi

**Affiliations:** 1 Research Institute for Medicines (iMed.ULisboa), Faculdade de Farmácia, Universidade de Lisboa, Av. Prof. Gama Pinto, 1649-003 Lisbon, Portugal; henrique.silva@campus.ul.pt; 2 Department of Pharmacy, Pharmacology and Health Technologies, Faculdade de Farmácia, Universidade de Lisboa, Av. Prof. Gama Pinto, 1649-003 Lisbon, Portugal; 3 Biophysics and Biomedical Engineering Institute (IBEB), Faculdade de Ciências, Universidade de Lisboa, Campo Grande, 1749-016 Lisbon, Portugal; 4 Institute of Physiology, Faculty of Medicine, University of Ljubljana, 1000 Ljubljana, Slovenia; js1716@student.uni-lj.si

**Keywords:** laser Doppler flowmetry, wavelet transform, microcirculation, oral glucose load, flowmotion, post-occlusive reactive hyperemia

## Abstract

**Simple Summary:**

There is increasing evidence to suggest that microcirculation becomes dysfunctional earlier than large blood vessels or the heart in several diseases. In diabetes mellitus, a disease characterized by chronic hyperglycemia, microvascular impairment is well-established; on the contrary, the effect of acute hyperglycemia in microcirculation remains unclarified. Our aim was to investigate the microvascular effect of an oral glucose load (OGL) using laser Doppler flowmetry (LDF) as a perfusion quantification technique, coupled with wavelet transform (WT) to perform a spectral decomposition of the LDF signal. On two distinct occasions (pre-load and post-load), sixteen healthy subjects drank either a standard glucose solution or water. Perfusion was assessed by LDF and WT while resting and during post-occlusive reactive hyperemia (PORH), evoked by a transient three-min occlusion of the brachial artery, in the forearm and the finger pulp. The OGL affected microcirculation in both sites compared to water, significantly blunting the PORH response in the forearm. The WT revealed significant differences in the cardiac and sympathetic components after OGL between the pre-load and post-load periods. These results suggest that an OGL induces a short-term subtle microvascular impairment, probably involving a modulation of the sympathetic nervous system.

**Abstract:**

Microcirculation in vivo has been assessed using non-invasive technologies such as laser Doppler flowmetry (LDF). In contrast to chronic hyperglycemia, known to induce microvascular dysfunction, the effects of short-term elevations in blood glucose on microcirculation are controversial. We aimed to assess the impact of an oral glucose load (OGL) on the cutaneous microcirculation of healthy subjects, quantified by LDF and coupled with wavelet transform (WT) as an interpretation tool. On two separate occasions, sixteen subjects drank either a glucose solution (75 g in 250 mL water) or water (equal volume). LDF signals were obtained in two anatomical sites (forearm and finger pulp) before and after each load (pre-load and post-load, respectively), in resting conditions and during post-occlusive reactive hyperemia (PORH). The WT allowed decomposition of the LDF signals into their spectral components (cardiac, respiratory, myogenic, sympathetic, endothelial NO-dependent). The OGL blunted the PORH response in the forearm, which was not observed with the water load. Significant differences were found for the cardiac and sympathetic components in the glucose and water groups between the pre-load and post-load periods. These results suggest that an OGL induces a short-term subtle microvascular impairment, probably involving a modulation of the sympathetic nervous system.

## 1. Introduction

Microcirculation in vivo has mostly been investigated in the skin by non-invasive optical technologies, such as laser Doppler flowmetry (LDF) and laser Doppler imaging, as well as photoplethysmography and polarized light spectroscopy [1]. Nevertheless, laser Doppler-based techniques have mostly been used due to their sensitivity and versatility [2,3]. The signal obtained by LDF is explained by interactions of impingent light with the moving red blood cells (RBC), which cause a wavelength shift, the well-known Doppler effect [4], and the blood flow is assessed by LDF as the product between the velocity of the moving RBC and their concentration.

Blood flow signals measured by LDF are known to be complex and multiscaled, resulting from contributions of several factors. Specifically, these flow signals receive inputs from the cardiac ejection and breathing cycles, as well as from local myogenic activity, post-ganglionic sympathetic terminals and from the endothelium, each of these phenomena being characterized by their own frequency range of activity, which is reflected in microvascular blood flow [5,6,7]. The cardiac component [ranging from 2.0–0.4 Hz] results from the transmission of cardiac pumping-related oscillations to the microvasculature; the respiratory component [0.4–0.15 Hz] results from the transmission of ventilation-related oscillations to the microvasculature, the myogenic component [0.15–0.052 Hz] from the intrinsic changes in the tone of vascular smooth muscle, the sympathetic component [0.052–0.021 Hz] from the control exerted on vascular smooth muscle by post-ganglionic sympathetic fibers, and the endothelial components are related to the secretion of endothelial vasodilators: nitric oxide (NO), reflecting the NO-dependent (NOd) [0.021–0.0095 Hz] and non-NO (i.e., NO-independent, NOi) [0.0095–0.0050 Hz] vasoactive mediators, such as prostacyclin, and other endothelium-derived hyperpolarizing factors.

Each component of the raw LDF signal can be individually assessed after performing spectral decomposition by appropriate mathematical tools such as wavelet (WT), or Fourier (FT) transforms [6,8]. A wavelet is defined as a small wave or oscillation of quick decay that can adopt different shapes depending on the goal of each analysis [9,10]. After choosing the shape of the original or “mother” wavelet, a family of wavelets is then obtained by stretching and shortening the mother wavelet’s length in time. Compressed wavelets are useful for the characterization of high-frequency phenomena, whereas low-frequency phenomena are characterized by dilated wavelets. For the assessment of blood flow signals, the Morlet wavelet has shown good localization in both time and frequency domains, as well as good correlation between time, width and corresponding frequency [9]. The WT can assess each individual component of a complex signal in terms of spectral location (i.e., frequency) and contribution to the overall signal (i.e., amplitude), thus allowing a mechanistic interpretation of the dynamics of perfusion regulation [11]. This tool has been successfully used in the past for characterizing microvascular dysfunction in several cardiovascular and metabolic diseases, such as hypertension [12], peripheral vascular disease [13], ischemic heart disease [14], and diabetes mellitus [15,16]. The WT shows several advantages over the FT, a classic spectral decomposition tool usually applied to blood flow signals. In particular, the WT provides a good localization in both time and frequency domains, whereas the FT only provides good localization in the frequency domain. Additionally, the WT shows a good performance in the analysis of non-stationary signals, such as those obtained after challenging microcirculation with provocation tests to assess its reactivity, whereas the use of FT is restricted to stationary signals [17].

It has been established that in several cardiovascular and metabolic diseases, microcirculation is rendered dysfunctional much earlier than large vessels or the heart [18,19]. Therefore, an appropriate knowledge of the mechanisms governing the microvascular function and dysfunction may help to prevent the progression or even the installation of certain diseases with an important vascular impact. Microvascular dysfunction is among the most important consequences of long-term hyperglycemia, one of the main features of diabetes mellitus, being characterized by impaired endothelium-dependent vasodilation [20], impaired vasomotion [21,22] and neurogenic regulation [23], together with increased capillary permeability [20].

Although chronically elevated blood glucose levels are known to induce microvascular dysfunction, it is unclear whether or not acutely elevated blood glucose levels affect microcirculation and if so, to what extent and (ir)reversibility. Recent studies have suggested that increased blood glucose levels during an oral glucose load (OGL) are associated with increased aortic stiffness and maladaptive carotid remodeling [24]. However, it remains underexplored whether short term elevations in blood glucose levels have an impact on microcirculation in healthy subjects; studies assessing this issue are scarce and their findings are partly contradictory [25,26,27]. Therefore, our objective was to assess the impact of a standard OGL on cutaneous microcirculation and its reactivity in young healthy subjects using LDF as a recording technique, coupled with WT as an interpretation tool, and performing post-occlusive reactive hyperemia (PORH) as a challenge to evaluate microvascular reactivity [28,29,30].

## 2. Materials and Methods

### 2.1. Subjects

Sixteen young healthy subjects, nonsmokers (21.4 ± 1.3 years old, 11 males, 5 females who were all in the follicular phase of their menstrual cycles) were enrolled in this study after informed written consent had been obtained. The study was approved by the local ethics committee (no. 0120-175/2017/6) and followed the recommendations of the Helsinki declaration and subsequent amendments for studies conducted in human subjects [31].

Before designing the study protocol for the LDF assessment, a preliminary experimental set enrolling 12 healthy participants was carried out to determine the time point corresponding to the highest blood glucose concentration after an OGL, which would then be chosen as the appropriate time to assess microvascular reactivity. Blood glucose was assessed spectrophotometrically by a portable glucometer (HemoCue Glucose201+, HemoCue AB, Ängelholm, Sweden) before (baseline) and after a standard OGL (75 g of glucose dissolved in 250 mL water); capillary blood samples were taken every ten minutes during a two hours period. We found that the highest glucose concentration was achieved after 35 min (8.9 ± 1.2 mmol/L, compared to baseline: 4.9 ± 0.4 mmol/L); in all subjects, glucose remained elevated for at least 60 min after OGL.

### 2.2. Procedure

Subjects had fasted for 12 h and acclimatized to controlled room conditions (24 ± 1 °C) for 20 min while lying supine. Each subject underwent two protocols on two separate sessions, the test one and the control one, both of them performed between 8 a.m. and noon. In the test protocol, the subjects drank a standard glucose solution (75 g dissolved in 250 mL water—the solution used for a standard oral glucose tolerance test in the clinical setting), whereas in the control protocol they drank the same volume of water, both solutions being kept at room temperature. In each protocol, each subject underwent three capillary blood samplings to assess blood glucose concentration: before the corresponding load (glucose or water), 20 min after the load and 50 min after the load, which corresponded to the end of the experiment. In each protocol, LDF recordings were performed after the 20-min acclimatization period, before and 35 min after the intervention (glucose and water load, respectively) when the plasma glucose (after the glucose load) reached its highest concentration (as assessed in a preliminary experiment described above). In each protocol, the following consecutive LDF recordings were performed while participants were lying in a supine position: a 10 min baseline recording before any intervention (resting), a PORH recording before the load (pre-load recording), a 10 min baseline recording after the intervention (glucose/water load), and a PORH recording after the intervention (post-load recording). The glucose/water intervention took place after blood flow had returned to baseline values after the first PORH recording and remained stable for at least ten minutes. Each PORH recording lasted for 15 min and consisted of a five-min baseline phase, a challenge phase in which a transient three-min occlusion of the brachial artery was performed, and a recovery phase lasting an additional seven minutes. For each protocol, perfusion was assessed simultaneously in two sites: the volar surface of the forearm (site 1) and the middle finger pulp (site 2) of the left upper limb.

### 2.3. Instruments

Local blood flow was quantified by LDF using the Periflux 4001 Master/4002 Satellite system (Perimed, Järfälla, Sweden) and expressed in arbitrary units (AU). The LDF probe was attached to the cleaned skin with an adhesive strip. Simultaneously, skin temperature was traced by a Peritemp device (Perimed, Järfälla, Sweden), blood pressure was non-invasively measured at the digital artery of the right hand middle finger (Finapres, Ohmeda, Englewood, USA). Blood glucose was measured by HemoCue Glucose201+ (HemoCue AB, Ängelholm, Sweden) after the acclimatization period, 20 min after glucose/water load (i.e., before the LDF was assessed), and at the end of the protocol.

### 2.4. Analytical

Raw LDF signals (500 Hz sampling frequency) were imported to Matlab software (Mathworks R015, Mathworks, USA), downsampled to 38 Hz and processed with a Morlet wavelet transform (WT) toolbox [32]. As previously mentioned, a family of wavelets can be obtained by stretching or dilating the length of a “mother” wavelet in time, as defined by:

(1) $$\psi_{a,b}\left(t\right)=\frac{1}{\sqrt{\left|a\right|}}\psi \left(\frac{t-b}{a}\right)a,b\in R,a\neq 0$$

The parameter *a* is the scaling parameter or scale, and it measures the degree of compression. The parameter *b* is the translation parameter which determines the time location of the wavelet. If |*a*| < 1, then the wavelet in the above equation is the compressed version (smaller support in time-domain) of the mother wavelet and corresponds mainly to higher frequencies. On the other hand, when |*a*| > 1, then ψ_a,b_(t) has a larger time-width than ψ(t) and corresponds to lower frequencies. In other words, compressed wavelets are able to extract high-frequency phenomena in the analyzed signal, whereas low-frequency phenomena are extracted by dilated wavelets.

**Figure 1:**
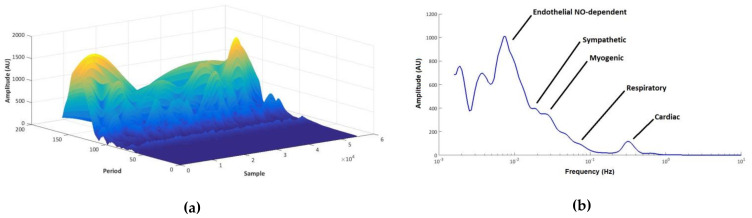
Wavelet transform of the laser Doppler flow (LDF) signal of a representative subject (resting period before the glucose load) in the time-frequency domain (three-dimensional) (**a**), and time-averaged (two-dimensional) (**b**). The typical frequencies of each component are shown as peaks in the time-frequency. Frequency is represented as the wavelet period on the time-frequency spectrum (**a**).

From the analysis of the raw LDF signals, frequency spectra were constructed for each study group (glucose load, water load) and for each period (before load, after load). The visual inspection of the spectra allowed the identification of the frequency intervals for each LDF component, whose contribution to the overall signal was assessed in relative terms as an amplitude ratio by dividing the area under the curve of each component by the area under the curve of the entire spectrum, and expressed as percentage. Typical three-dimensional and two-dimensional spectra obtained for a representative subject are depicted in Figure 1a,b, respectively.

After being processed with the WT, the portions of the LDF signals lying outside the “cone of influence” (i.e., a region outside of which the spectrum becomes distorted due to edge effects) were not considered for statistical analysis. For the resting recording, perfusion and the components’ amplitude ratios were calculated between 3 and 7 min. As for the PORH recordings, the following three analysis periods were considered: baseline from 3 to 5 min, hyperemia from 8 to 10 min (time to reach a stable perfusion after initiating reactive hyperemia), and recovery from 11 to 13 min.

All variables were obtained by the time-average in each respective interval and presented as the median and the corresponding limits of the 95% confidence interval (due to non-normal distribution). The Shapiro–Wilk test was used to assess the normality of the statistical distribution of the samples. The data of blood glucose concentration were found to be normally distributed, presented as mean and standard deviation (SD) and were compared with the t-test for related samples. However, all LDF-related variables were found to follow a non-normal statistical distribution, and therefore phase comparisons within each protocol (baseline vs. PORH; baseline vs. recovery) were carried out with the Wilcoxon signed-rank test. For comparisons between protocols (glucose load vs. water load for each phase) the Mann–Whitney U test for independent samples was used. *p* < 0.05 was defined as significant for all statistical tests. Statistical analyses were performed in SPSS 22.0 (IBM, USA) and graphical representation of data was carried out with Matlab and MS Excel 2010.

## 3. Results

**Figure 2:**
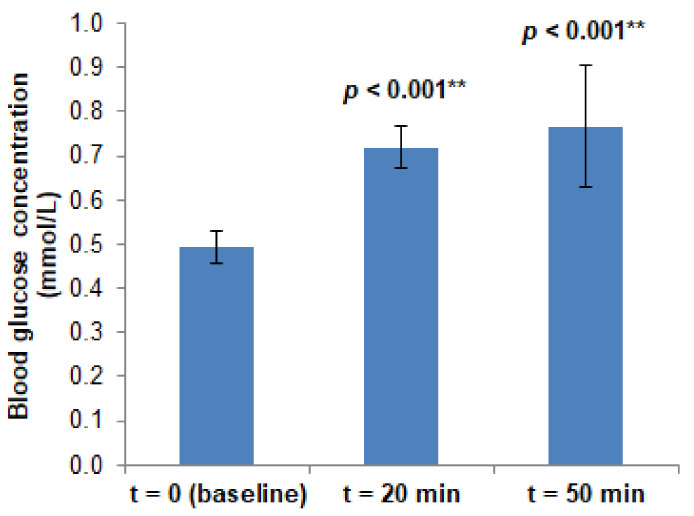
Blood glucose concentration after the OGL challenge at three different measurement points (t = 0 min, corresponding to the fasting state; t = 20 min; t = 50 min). Data are presented as the mean and SD; N = 16. Statistical comparisons with the baseline (t = 0 min) are presented (t-test for the paired samples; ** *p* < 0.01).

In all subjects, OGL induced a significant increase in blood glucose concentration that persisted at least 50 min after ingestion, corresponding to the end of the protocol (Figure 2), but the water load did not (data not shown). During either glucose or water load, no significant changes in blood pressure and skin temperature were observed (data not shown). Similarly, blood pressure remained stable during both PORH challenges. Therefore, these variables are not expected to have affected vascular conductance and lessened the meaning of the absolute values of the LDF signal, justifying our further analysis of the flow (rather than conductance) data.

### 3.1. Resting Blood Flow Recordings

**Figure 3:**
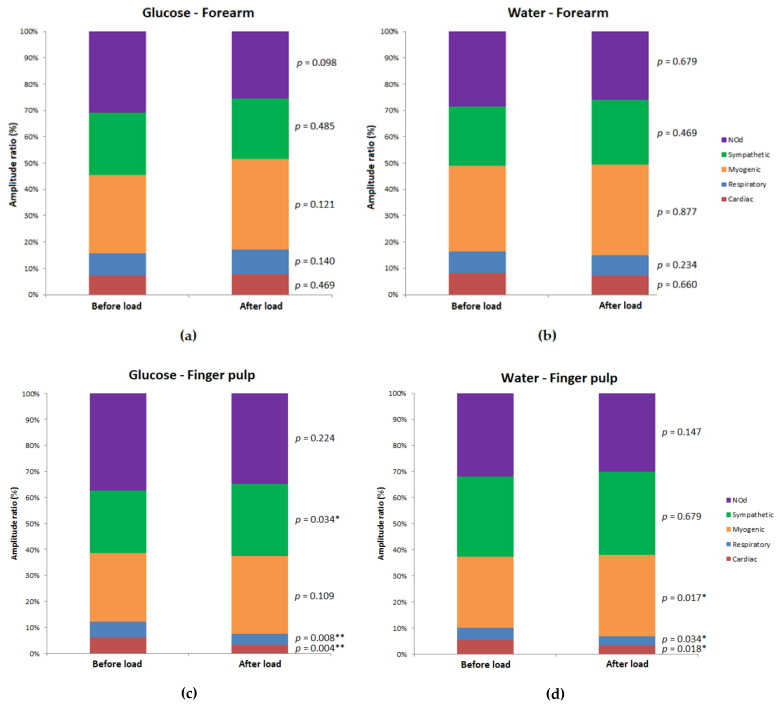
Relative contribution of the laser Doppler flow (LDF) signal components (i.e., amplitude ratio, expressed in percentage of the total LDF spectrum) before and after glucose and water load for the forearm (a and b, respectively) and the finger pulp (c and d, respectively) between 3 and 7 min of the 10-min resting recording; N = 16. Statistical comparisons are presented (Wilcoxon signed-rank test for related samples for the pre-load vs. post-load comparisons; * *p* < 0.05; ** *p* < 0.01).

The effects of glucose and water load on the resting blood flow recordings are presented in Table 1 and in Figure 3. In the forearm, neither glucose nor water load produced any significant changes in the LDF signal or in any of its components. Similarly, no significant differences were observed between the glucose or water groups, either in the pre-load or post-load periods.

However, in the finger pulp, the glucose load produced a significant decrease in the LDF signal (*p* = 0.004), and a decrease in the relative contribution of the cardiac (*p* = 0.004) and respiratory (*p* = 0.008) components to the whole LDF spectrum. However, it significantly increased the relative contribution of the sympathetic component (*p* = 0.034). Water load also decreased the LDF signal in the finger pulp significantly (*p* = 0.001), together with the cardiac (*p* = 0.018) and respiratory (*p* = 0.034) components, whereas the myogenic component increased significantly (*p* = 0.017). No significant differences were observed between the glucose and water groups either in the pre-load or post-load periods.

### 3.2. PORH Profile Recordings

#### 3.2.1. Forearm

**Figure 4:**
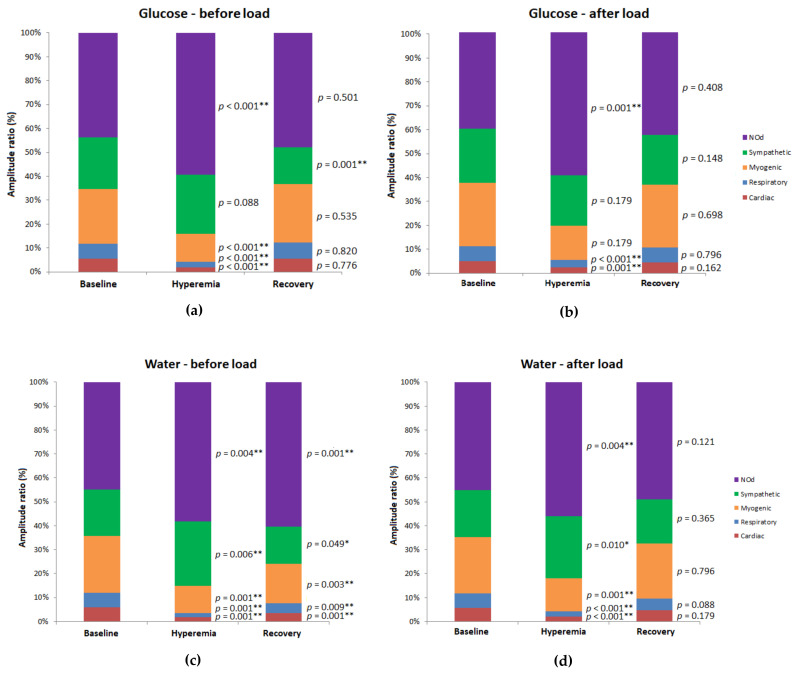
Relative contribution of the laser Doppler flow (LDF) signal components (i.e., amplitude ratio, expressed as a percentage of the total LDF spectrum) before and after glucose (**a** and **b**, respectively) and the water load (**c** and **d**, respectively) for the forearm in the different phases (3 to 5 min for the baseline; 8 to 10 min for hyperemia; 11 to 13 min for recovery) of the PORH protocol; N = 16. Statistical comparisons are presented (Wilcoxon signed-rank test for the related samples for the pre-load vs. post-load comparisons; ** *p* < 0.01).

The effects of glucose and water load on the PORH profile of the forearm skin are presented in Table 2 and in Figure 4. Before glucose load, a significant increase in the LDF signal after cuff release was observed (*p* = 0.001), the well-known reactive hyperemia phenomenon, which was accompanied by a significant decrease in the cardiac, respiratory and myogenic components (*p* < 0.001 for all) and by a significant increase in the NOd component (*p* < 0.001). The sympathetic component did not change significantly during hyperemia. During the recovery phase, the LDF signal remained significantly higher than baseline (*p* = 0.027), whereas the sympathetic component remained significantly lower (*p* = 0.001). No significant changes were detected for the remaining components during recovery.

However, after glucose load, the PORH profile changed slightly compared to before glucose load, especially in the recovery phase. Significant hyperemia was observed once again (*p* < 0.001), accompanied by a significant decrease in the relative contribution of the cardiac (*p* = 0.001), respiratory (*p* < 0.001), and myogenic (*p* = 0.001) components, and by a significant increase in the endothelial NOd component (*p* = 0.001), whereas the sympathetic component did not change significantly during this phase. However, during recovery, there was no significant difference either in the LDF signal or in any of the components compared to baseline.

After water load, the hyperemia (*p* < 0.001) was accompanied by a significant decrease in the cardiac (*p* < 0.001), respiratory (*p* < 0.001), and myogenic (*p* = 0.001) components. However, a significant increase in the sympathetic (*p* = 0.010) and NOd (*p* = 0.004) components was noted. During recovery, perfusion remained significantly higher than baseline (*p* = 0.005), while no significant change was observed for all components. No significant differences in the LDF signal were found between the glucose and water groups in the pre-load period for either phase of the PORH challenge. However, in the post-load period, the LDF signal was significantly lower during hyperemia in the glucose load than in the water load (*p* = 0.021). On the other hand, no differences were noted in the LDF signal for the baseline and recovery phases of PORH between glucose and water loads.

#### 3.2.2. Finger Pulp

**Figure 5:**
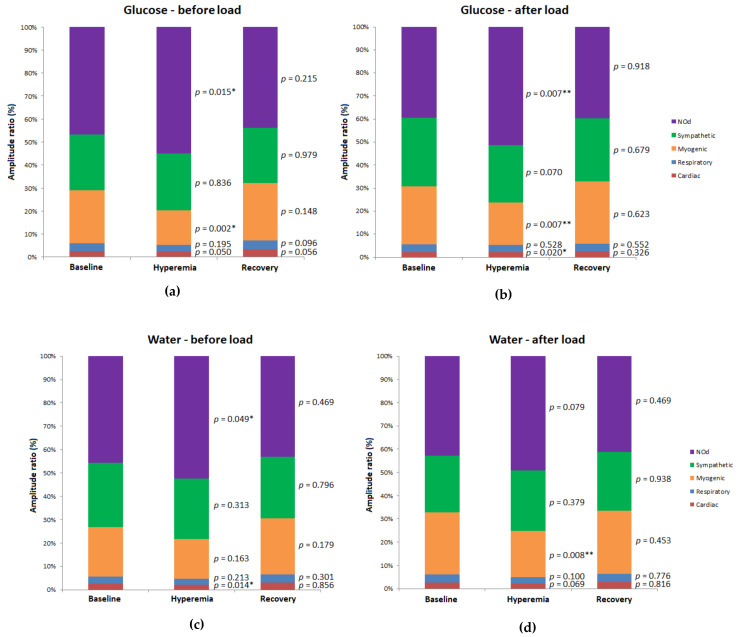
Relative contribution of the laser Doppler flow (LDF) signal components (i.e., amplitude ratio, expressed as a percentage of the total LDF spectrum) before and after the glucose (**a** and **b**, respectively) and water (**c** and **d**, respectively) loads for finger pulp in the different phases (3 to 5 min for the baseline; 8 to 10 min for hyperemia; 11 to 13 min for recovery) of the PORH protocol; N = 16. Statistical comparisons are presented (Wilcoxon signed-rank test for the related samples for the pre-load vs. post-load comparisons; * *p* < 0.05; ** *p* < 0.01).

The effects of glucose and water load on the PORH profile in the finger pulp are presented in Table 2 and in Figure 5. Before glucose load, reactive hyperemia (*p* = 0.013) was accompanied by a significant decrease in the myogenic component (*p* = 0.002) and by a significant increase in the NOd (*p* = 0.015) component. During recovery, no significant change regarding baseline was observed for blood flow or LDF components. After glucose load, the reactive hyperemia (*p* = 0.020) was accompanied by a significant decrease in the myogenic component (*p* = 0.007) and by a significant increase in the NOd (*p* = 0.007) components. During recovery, neither blood flow nor the components changed significantly. Before water load, the reactive hyperemia (*p* = 0.006) was accompanied by a significant decrease in the cardiac component (*p* = 0.014) and by a significant increase in NOd (*p* = 0.049). After water load, the reactive hyperemia (*p* = 0.004) was also accompanied by a significant decrease in the myogenic component (*p* = 0.008) but without other significant changes. Finally, no significant differences in the LDF signal were found between the glucose and water loads for either phase of the pre-load or post load period of the PORH challenge.

The impact of the glucose and water loads on the PORH challenge was quantified as a variation (LDF value in the post-load period minus LDF value in the pre-load period). This variation was statistically compared between the glucose and water groups, as shown in Table 3. In the forearm, statistical differences between glucose and water groups were observed in the hyperemia and recovery phases. The LDF signal during hyperemia increased between pre-load and post-load periods with water (median 4.7 AU), whereas it decreased with glucose (median −1.7 AU), a change that exhibited a significant difference (*p* = 0.001). During recovery, despite no changes in the LDF signal being found, the cardiac component decreased between pre-load and post-load periods with glucose (median −1.1 AU), whereas it increased with water (median 0.6 AU), which revealed statistical significance (*p* = 0.001).

In the finger pulp, the LDF signal did not differ significantly between the pre-load and post-load periods with either glucose or water. However, the relative contribution of the sympathetic component to the whole LDF spectrum increased with glucose (median 3.7 AU) whereas it decreased with water (median −3.7 AU), a difference exhibiting statistical significance (*p* = 0.003).

## 4. Discussion

Our results have shown that a standard OGL induces short-term effects on cutaneous microcirculation, which was revealed during a PORH challenge. Specifically, during hyperemia the LDF signal was significantly lower with glucose than with water in the post-load period. Moreover, the LDF signal exhibited opposite profiles between the pre-load and post-load periods, having decreased with glucose and increased with water. In contrast, a glucose load-mediated effect was not evident from the resting LDF recordings. This highlights the need to employ challenge tests such as the PORH challenge to increase the sensitivity of the LDF technique for microvascular assessment [28]. By applying the WT we have shown for the first time that acute OGL induces changes in the spectral components of the LDF signal implying a modulation of the sympathetic influence on microvascular regulation.

From the analysis of the raw LDF signal, the blunted hyperemia response after OGL was only observed in the forearm, but not in the finger pulp. This suggests that the microvascular effect of a glucose load is site-specific. The anatomical differences and the differences in the regulation of microvascular flow in terms of sympathetic innervation, and the contribution of endothelial factors seem to partly explain our observation. The forearm has been described to show lower reproducibility than the finger pulp with regard to the PORH response, which has been attributed to considerable anatomical variability [28]. In fact, the finger pulp contains more arteriovenous anastomoses, which also implies a richer innervation by post-ganglionic sympathetic fibers that are important for thermoregulation responses [18,19,33]. Considering the fact that both ingested solutions (glucose and water) were kept at room temperature, being quite lower than the core temperature, it is admissible that thermoregulation mechanisms may have been evoked. Specifically, as both glucose and water induced a similar decrease in the resting LDF signal and exhibited an overall similarity of PORH in the pulp (but not in the forearm), a thermoregulation-mediated sympathetic activation may have occurred and induced a redistribution of blood flow from the skeletal muscle and skin vascular beds towards the celiac and mesenteric circulations [34], which is likely to be more evident in the sympathetically rich finger pulp. Besides this thermoregulatory response, it is possible that increased blood concentration of insulin might have contributed to increased sympathetic drive, as previously observed in humans [35].

To the best of our knowledge, this is the first study that decomposed LDF signals by the WT to gain insight into the physiological mechanisms underlying the effect of an OGL on cutaneous microcirculation. All LDF spectra revealed five main frequency components [36]—cardiac [33], respiratory [37], myogenic (i.e., vasomotion) [38], sympathetic [39], and endothelial activity, the latter being subdivided into NOd and NOi [36]. These components showed no significant frequency shifts (i.e., no shifts of the frequency corresponding to the maximal amplitude; data not shown) with either glucose or water load in either pre-load or post-load periods. However, the time length of our LDF signals was not extensive enough to allow a clear identification of the NOi component (0.095–0.005 Hz), which may have prevented gaining a full insight into the currently discussed physiological mechanisms. In previously published studies the contribution of each component of the LDF signal has been expressed either in terms of absolute [8,9] or relative values [11]. To minimize the impact of inter-subject variability in terms of raw LDF signals and the corresponding frequency spectra, we believe that expressing the contribution of each component in relative values is more appropriate.

Our results obtained by applying the WT highlight differences in the spectra obtained in different anatomical sites during resting and PORH recordings. In the resting recordings, glucose and water load significantly affected the finger pulp but not the forearm skin, the opposite of what was observed for PORH. Glucose and water load increased the relative contribution of the myogenic and sympathetic components, but only significantly for the former. The relative increase in these low-frequency components seems to explain the significant decrease in the relative contributions of the high-frequency cardiac and respiratory components. An increase in peripheral sympathetic drive has already been demonstrated to occur after both oral glucose [40] and water [41] loads, yet by applying tests different than WT. This parallel increase in the sympathetic and myogenic components might derive from the fact that the sympathetic drive also potentiates the myogenic activity [42]. The NOd component, in contrast, was little affected by either glucose or water load. This result was not expected given that an increase in blood glucose concentration increases the secretion of insulin, which has been implicated to potentially affect the release of endothelial vasodilators, namely NO [43], whose contribution to the regulation of vascular tone is also site dependent [44]. Furthermore, glucose also displays a negative effect on NO synthase activity through a mechanism involving activation of protein kinase C [45]. Considering the existence of a cross-talk between different local microvascular components [46], the different extent to which insulin might act on the cutaneous microvasculature in each subject also seems to have contributed to different impact on microcirculation in different sites.

Even though the analysis of the raw LDF signals showed a blunted PORH response with glucose in the forearm but not in the finger pulp, the WT analysis revealed that several LDF components were affected in both sites (Table 2 and Table 3), and differently compared to the resting conditions. This is in line with the concept of PORH that has been used to challenge microcirculation and evaluate its function in clinical practice, particularly the endothelium-dependent vasodilation [44].

The decrease in the myogenic component in the forearm during the pre-load glucose period suggests a reduction in vasomotion amplitude resulting from vessel dilation during rapid refilling following occlusion release [47]. The increase in NOd could be attributed to a compensatory increase of endothelial NO release in response to decreased oxygen partial pressure in the tissues [47,48] and to increased shear stress during hyperemia after release of the occlusion. In fact, both myogenic and endothelial responses are thought to constitute the main mechanisms underlying PORH [47]. The decrease in the high-frequency cardiac and respiratory components reflects a decreased transmission of cardiac and ventilation-derived pulses to the peripheral vasculature. During recovery, the LDF signal was still significantly higher than baseline, again due to the myogenic response, which increased towards the baseline (Figure 4) [49]. In addition, a decrease in the sympathetic component was observed, also favoring an increase in the LDF signal, a mechanism probably related to the relieving of pain perception during occlusion release.

The raw LDF signal showed a slightly blunted PORH response after the glucose load. Our WT analysis helped to clarify potential mechanisms responsible for these slight differences (Figure 4). During hyperemia, the NOd component showed a significant increase compared to baseline, whereas the cardiac and respiratory components decreased significantly. However, the myogenic component did not change during this phase, which contrasts with the pre-load period. Furthermore, during recovery the sympathetic component showed no difference compared to baseline. These results suggest that OGL affected the PORH response due to a combined modification of the sympathetic and myogenic components. In contrast, the hyperemic response observed after water load was identical for both pre-load and post-load periods. The main difference was that in the post-load period no significant differences were found between recovery and baseline. Our results seem to suggest that the PORH profiles obtained after glucose and water are significantly different, either in the pre-load or post-load periods, apparently undermining our analysis. However, when the differences between each component between the pre-load and post-load periods were compared, an opposite profile for the cardiac component was observed during recovery (i.e., an increase with glucose and a decrease with water). Considering that the cardiac component reflects mainly the transmission of the arterial pulse to the microvascular bed, this observation is in line with the profile previously observed for the raw LDF signal during hyperemia.

Mechanisms underlying the PORH response in the finger pulp appear to be different to the ones regarding the forearm (Figure 5), suggesting that glucose produced no changes in microvascular reactivity at this site. Again, slight differences in the pre-load period were found between glucose and water. Overall, these results again suggest a low reproducibility of the PORH challenge test, especially when making direct comparisons between variables.

For this reason, the differences between the pre-load and post-load periods were compared, revealing significant differences between glucose and water in the sympathetic component (i.e., a decrease with water and an increase with glucose) during baseline (Table 3). This opposite tendency highlights that an increase in the sympathetic drive, either due to thermoregulation or to insulin secretion, might have been induced by OGL in this anatomical site, which is also in line with the baseline recordings obtained after OGL.

Taken together, our results seem to suggest that OGL affects cutaneous perfusion in healthy subjects to a slight degree, and differently so in distinct anatomical sites. In the present experimental conditions, a potential impact of an acute glucose load on microcirculation, even though discrete, seems to have been detected by the WT, highlighting the usefulness of this analytical tool for flowmotion analysis. Nevertheless, the interpretation of data obtained by the WT is still far from understood and warrants further studies as well as analytical improvements. Effectively, the existence of a cross-talk between different components of microcirculation cannot be ignored, and their implications in the interpretation of WT spectra are yet to be clarified.

## 5. Limitations

The relatively small sample size and asymmetrical distribution of male and female subjects constitute a limitation. All subjects performed the protocol in the same order, starting with the glucose load challenge, and the LDF signals were consistently acquired in the left upper limb. This lack of randomization in terms of the order of the challenge and in terms of limb choice also constitute potential limitations. The raw LDF signals and corresponding WT spectra showed, as expected, considerable inter-subject variability. This prevented the assessment of the components’ activity in terms of absolute values, which the authors regard as a limitation. In order to decrease this variability, the activity of the LDF components was consistently expressed in terms of relative values. Finally, we were not able to assess the relative contribution of the NOi component to microvascular reactivity since our time series were much too short. This limitation prevents the corroboration of more thorough mechanisms of microvascular regulation.

## 6. Conclusions

This study is the first to combine cutaneous perfusion quantification assessed by LDF with spectral decomposition performed by WT to more sensitively assess the microvascular response to an OGL. Our results suggest that acute OGL affects the overall PORH profile by altering the WT spectra of the LDF signals. Minor alterations in various spectral components of the LDF signal were found, suggesting that OGL impacts microvascular reactivity in a subtle way, potentially contributing to microvascular dysfunction. Differences in the behavior of the LDF spectra between the finger pulp and the forearm after glucose (and water) load expose different control mechanisms in these very sites and different reactivity after OGL. Additional studies should further explore the role of the sympathetic nervous system as well as a potential role of insulin in acute microvascular effects of an OGL in healthy subjects.

## Figures and Tables

**Table 1:** Laser Doppler flowmetry (LDF) signal of the resting recording, obtained at the forearm and the finger pulp. LDF signal (expressed in arbitrary units) is presented as the median and the limits of the 95% confidence interval (CI). Variables are presented for pre-load and post-load periods, for glucose (G, test) and water (W, control). Statistical comparisons are presented (Wilcoxon signed-rank test for related samples for the before load vs. after load comparisons; Mann–Whitney U test for independent samples for the glucose vs. water comparisons; ** *p* < 0.01).

Site	Group			Pre-Load	Post-Load	*p* Value (Post-Load vs. Pre-Load)
Forearm	Glucose (test)	Median		5.6	6.0	0.660
		95% CI	Upper	9.4	9.9	
			Lower	4.6	5.3	
	Water (control)	Median		7.1	7.5	0.173
		95% CI	Upper	8.8	9.2	
			Lower	6.3	6.5	
	*p* value (G vs. W)			0.138	0.254	-
Finger pulp	Glucose (test)	Median		260.1	221.9	0.004 **
		95% CI	Upper	351.8	287.5	
			Lower	221.0	160.4	
	Water (control)	Median		312.1	238.6	0.001 **
		95% CI	Upper	328.1	264.1	
			Lower	244.3	159.4	
	*p* value (G vs. W)			0.616	0.985	-

**Table 2:** Laser Doppler flowmetry (LDF) signal during the post-occlusive reactive hyperemia (PORH) on the forearm and finger pulp before and after the glucose/water loads. The median and the 95% confidence interval (CI) limits of the LDF signal (expressed as arbitrary units) in three PORH phases (baseline, hyperemia, recovery) before and after the glucose (test) and water (control) loads are presented. Statistical comparisons are presented (Wilcoxon signed-rank test for the related samples for the hyperemia vs. the baseline and recovery vs. the baseline comparisons; Mann Whitney U test for the independent samples for the glucose vs. water comparisons; * *p* < 0.05; ** *p* < 0.01).

				Before Load					After Load				
Site	Group	Parameter		Bas.	Hyper.	Rec.	*p* Value (Hyper. vs. Bas.)	*p* Value (Rec. vs. Bas.)	Bas.	Hyper.	Rec.	*p* Value (Hyper. vs. Bas.)	*p* Value (Rec. vs. Bas.)
Forearm	glucose (test)	median		6.0	12.8	6.2	0.001 **	0.027 *	6.2	11.0	6.7	< 0.001 **	0.244
		95% CI	upper	8.3	17.3	8.2			9.5	8.9	9.7		
			lower	5.1	11.5	5.5			5.4	15.9	5.6		
	water (control)	median		7.5	11.5	7.9	0.004 **	0.001 **	7.4	19.3	8.3	< 0.001 **	0.005 **
		95% CI	upper	9.0	18.8	10.7			9.3	24.4	10.0		
			lower	6.1	9.3	6.7			6.3	14.7	6.8		
		*p* value (G vs. W)		0.287	0.468	0.094	-	-	0.468	0.021 *	0.224	-	-
Finger pulp	glucose (test)	median		249.2	288.7	228.8	0.013 *	0.056	203.8	249.1	198.5	0.020 *	0.326
		95% CI	upper	333.3	373.7	321.1			281.6	303.0	281.3		
			lower	215.0	245.2	202.5			141.1	216.7	145.9		
	water (control)	median		313.8	325.8	306.8	0.006 **	0.408	257.4	295.6	269.2	0.004 **	0.301
		95% CI	upper	332.4	354.6	317.7			266.4	320.3	279.5		
			lower	223.7	266.8	214.2			155.9	242.6	178.4		
		*p* value (G vs. W)		0.539	0.468	0.468	-	-	0.642	0.287	0.361	-	-

**Table 3:** Laser Doppler flow (LDF) differences and differences in the corresponding LDF components before and after the glucose (and water) load. The median and limits of the 95% confidence interval (CI) of variation (after load – before load) on the forearm and finger pulp in the different phases (baseline, hyperemia, recovery) of the PORH protocol are presented. Statistical comparisons are shown (Mann Whitney U test for the independent samples for the glucose vs. water comparisons; 0.05; ** *p* < 0.01).

LDF Signal/LDF Components				LDF Signal Variation (Post-Load–Pre-Load)					
				Forearm			Finger Pulp		
				Baseline	Hyperemia	Recovery	Baseline	Hyperemia	Recovery
Raw LDF signal	Glucose	median		0.2	−1.7	0.5	−52.7	−33.2	−31.7
		95% CI	upper	2.5	0.6	1.9	−24.2	−92.5	−0.8
			lower	−1.1	−4.7	−0.4	−101.5	−6.8	−95.7
	Water	median		0.4	4.7	0.6	−38.8	−39.3	−38.8
		95% CI	upper	1.0	9.7	1.0	−21.8	−3.2	6.8
			lower	−0.6	1.2	−1.6	−122.6	−55.2	−81.0
	*p* value (G vs. W)			0.752	0.001 **	0.539	0.780	0.780	1.000
Card	Glucose	median		−0.4	0.5	−1.1	−0.3	0.1	−0.6
		95% CI	upper	0.4	0.7	−0.3	0.1	0.2	−0.1
			lower	−1.3	−0.2	−1.8	−1.0	−0.5	−1.5
	Water	median		−1.0	0.4	0.6	−0.1	−0.1	−0.2
		95% CI	upper	1.6	1.2	2.1	0.5	0.3	0.6
			lower	−2.1	−0.5	0.2	−0.6	−0.4	−1.3
	*p* value (G vs. W)			0.564	0.515	0.001 **	0.468	0.838	0.224
Resp	Glucose	median		0.6	0.3	0.5	−0.3	0.4	−0.4
		95% CI	upper	1.4	1.3	−0.1	1.7	1.2	1.8
			lower	−1.4	−0.1	−1.1	−1.6	−0.5	0.1
	Water	median		−0.3	0.5	1.3	0.3	0.1	−0.1
		95% CI	upper	0.7	0.7	1.1	1.2	0.8	0.9
			lower	−0.5	−0.3	−2.1	−0.2	−0.3	−0.8
	*p* value (G vs. W)			0.445	0.838	0.138	0.381	0.956	0.160
Myo	Glucose	median		3.6	1.5	2.9	2.4	2.9	2.6
		95% CI	upper	5.5	5.6	5.9	6.2	4.7	5.1
			lower	0.7	−0.3	−2.4	−1.6	1.5	−1.0
	Water	median		−1.6	3.0	8.0	4.9	3.1	3.3
		95% CI	upper	4.8	6.1	10.7	9.2	6.2	7.7
			lower	−5.1	−1.2	2.3	1.6	−0.2	−1.0
	*p* value (G vs. W)			0.160	0.724	0.094	0.224	0.752	0.696
Symp	Glucose	median		2.7	−4.3	5.9	3.2	1.2	3.7
		95% CI	upper	3.7	0.9	8.2	9.8	3.1	7.5
			lower	−1.6	−9.9	1.6	0.9	−2.4	−0.5
	Water	median		0.0	−3.4	1.9	−3.7	−0.3	0.6
		95% CI	upper	3.9	4.6	6.8	0.2	4.5	2.7
			lower	−3.5	−6.1	−1.0	−6.6	−4.4	−5.5
	*p* value (G vs. W)			0.305	0.564	0.110	0.003 **	0.724	0.080
NOd	Glucose	median		−6.4	−1.8	−6.8	−7.3	−4.4	−2.7
		95% CI	upper	0.1	4.5	−0.7	−0.7	0.3	0.8
			lower	−7.6	−6.4	−9.7	−13.8	−7.4	−9.2
	Water	median		2.5	0.2	−11.8	−3.5	−3.6	−1.3
		95% CI	upper	6.4	5.6	−4.1	3.0	3.6	3.6
			lower	−6.1	−10.4	−18.9	−8.3	−10.1	−6.9
	*p* value (G vs. W)			0.119	0.780	0.110	0.171	0.539	0.669

## Data Availability

The data presented in this study are available on request from the corresponding author.
